# Deep learning driven segmentation of maxillary impacted canine on cone beam computed tomography images

**DOI:** 10.1038/s41598-023-49613-0

**Published:** 2024-01-03

**Authors:** Abdullah Swaity, Bahaaeldeen M. Elgarba, Nermin Morgan, Saleem Ali, Sohaib Shujaat, Elena Borsci, Israel Chilvarquer, Reinhilde Jacobs

**Affiliations:** 1 OMFS IMPATH Research Group, Department of Imaging and Pathology, Faculty of Medicine, KU Leuven, & Department of Oral and Maxillofacial Surgery, University Hospitals Leuven, Leuven, Belgium; 2 Prosthodontic Department, King Hussein Medical Center, Jordanian Royal Medical Services, Amman, Jordan; 3 Department of Prosthodontics, Tanta University, Tanta, Egypt; 4 Department of Oral Medicine, Faculty of Dentistry, Mansoura University, Mansoura, Egypt; 5 Restorative Dentistry Department, King Hussein Medical Center, Jordanian Royal Medical Services, Amman, Jordan; 6 King Abdullah International Medical Research Center, Department of Maxillofacial Surgery and Diagnostic Sciences, College of Dentistry, King Saud Bin Abdulaziz University for Health Sciences, Ministry of National Guard Health Affairs, Riyadh, Kingdom of Saudi Arabia; 7 Oral Diagnostic Clinic, Karolinska Institute, Stockholm, Sweden; 8 Department of Oral Radiology, School of Dentistry, University of São Paulo (USP), São Paulo, Brazil; 9 Department of Dental Medicine, Karolinska Institute, Stockholm, Sweden

**Keywords:** Cone-beam computed tomography, Digital radiography in dentistry, Dentistry, Orthodontics

## Abstract

The process of creating virtual models of dentomaxillofacial structures through three-dimensional segmentation is a crucial component of most digital dental workflows. This process is typically performed using manual or semi-automated approaches, which can be time-consuming and subject to observer bias. The aim of this study was to train and assess the performance of a convolutional neural network (CNN)-based online cloud platform for automated segmentation of maxillary impacted canine on CBCT image. A total of 100 CBCT images with maxillary canine impactions were randomly allocated into two groups: a training set (n = 50) and a testing set (n = 50). The training set was used to train the CNN model and the testing set was employed to evaluate the model performance. Both tasks were performed on an online cloud-based platform, ‘Virtual patient creator’ (Relu, Leuven, Belgium). The performance was assessed using voxel- and surface-based comparison between automated and semi-automated ground truth segmentations. In addition, the time required for segmentation was also calculated. The automated tool showed high performance for segmenting impacted canines with a dice similarity coefficient of 0.99 ± 0.02. Moreover, it was 24 times faster than semi-automated approach. The proposed CNN model achieved fast, consistent, and precise segmentation of maxillary impacted canines.

## Introduction

The maxillary canine is the second most frequently impacted tooth, characterized by the failure of a canine to emerge through the gingiva and assume its correct position following the anticipated eruption time. This is due to the fact that it is often the last tooth to erupt and has a long pathway from its developmental position deep within the maxilla to its final location in the oral cavity^1^. Maxillary canine impaction occurs in approximately 2% of the population (range from 1.7 to 4.7%), with a higher prevalence in females than males^2^. Several etiological factors might contribute to its impaction, including genetic factors, lack of space, tooth root developmental abnormalities, trauma or injury and presence of oral pathological lesions^3,4^.

The proper positioning of maxillary canine in the dental arch is critical for functional occlusion^5^ and aesthetics^1,6^. A delayed diagnosis or lack of treatment can result in complications such as midline shift, tooth displacement, arch length defect, ankylosis, follicular cyst development, internal tooth resorption, pain, caries, and recurrent infection^7^. Hence, early detection and intervention are crucial. The diagnosis of canine impaction and determination of the appropriate treatment plan necessitates the utilization of radiographic imaging in conjunction with patient history and clinical examination. In this context, cone beam computed tomography (CBCT) is the most optimal radiographic imaging tool due to its ability to accurately determine the tooth’s three-dimensional (3D) position and assess its relationship with the neighboring teeth and other neighboring anatomical structures. This enables clinicians to accurately assess potential treatment options and plan the most effective course of action^8–11^.

Recently, the field of oral healthcare has witnessed a shift towards the utilization of digital workflows for diagnostic and treatment planning purposes. These workflows have addressed the shortcomings of conventional methods by offering enhanced precision, time-efficiency, and improved patient care^12,13^. The implementation of such workflows has facilitated patient-specific virtual planning, orthodontic treatment simulation, treatment progress monitoring, and 3D printing of orthodontic appliances^14–16^. This could prove particularly advantageous for complex treatment procedures such as those involving impacted canines.

In digital dental workflows involving impacted canines, CBCT image segmentation is a crucial initial step for creating an accurate 3D model of the tooth for either diagnosis, planning or outcome assessment. Any error at this stage can adversely affect the final result^17^. Both manual and semi-automated segmentation (SS) have been applied as clinical standards for creating virtual impacted canine models, where manual segmentation is time-consuming and operator dependent^18,19^. Meanwhile, SS relies on threshold selection and often requires manual adjustments, which also makes it prone to human error^20,21^. Recent application of deep convolutional neural networks (CNNs) has demonstrated improved performance over conventional segmentation methods for modeling of the dentomaxillofacial region, with promising results for automated segmentation (AS) of teeth, upper airway, inferior alveolar nerve canal, mandible, and maxillary sinus on CBCT images^22–30^. However, there is a lack of evidence regarding the application of CNNs for the AS of impacted canines.

Therefore, the aim of the present study was to train and assess the performance of a CNN-based tool for AS of maxillary impacted canine on CBCT images.

## Material and methods

This retrospective study was conducted in compliance with the World Medical Association Declaration of Helsinki on medical research. Ethical approval was obtained from the Ethical Review Board of the University Hospitals Leuven (reference number: B322201525552).

### Dataset

A total of 200 CBCT scans (46 males and 54 females; age range: 8–54 years) having uni- or bilateral maxillary impacted canine cases were collected during the period 2015–2022, from the radiological database of UZ Leuven Hospital, Leuven, Belgium. Inclusion criteria consisted of previously clinically and radiologically diagnosed unilateral/bilateral, horizontal/oblique/vertical and complete/partial maxillary canine impactions. Teeth with both complete and partially formed roots were included. The majority of cases in these datasets had orthodontic brackets. Exclusion criteria involved scans with motion artifacts and poor image quality, where margins of canine could not be optimally delineated. The CBCT images were obtained utilizing two devices, NewTom VGi Evo (Cefla, Imola, Italy) and 3D Accuitomo 170 (J Morita, Kyoto, Japan) with variable scanning parameters of 90–110 kV, a voxel size between 0.125 and 0.300 mm^3^ and a field of view between 8 × 8 and 24 × 19 cm.

**Figure 1:**
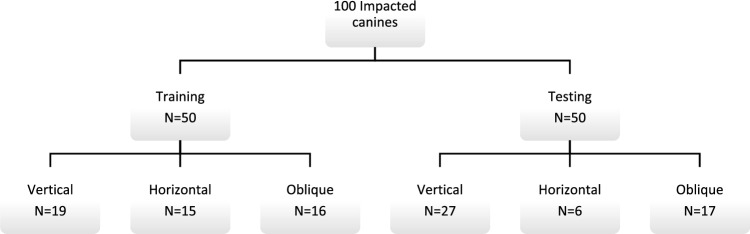
Dataset used for training and validation.

All images were exported in Digital Imaging and Communications in Medicine (DICOM) format. Thereafter, the DICOM datasets were uploaded to a CNN-based online cloud platform known as the ‘Virtual patient creator’ (Relu, Leuven, Belgium), to assess if the tool would be able to segment impacted canines, as it had been previously trained for permanent erupted teeth segmentation^24,28^. Based on the visual assessment by two observers (A.S, B.E), 100 images from the total dataset of 200 images could not be segmented automatically by the platform. Hence, these failed cases were randomly divided into two subsets, training set (n = 50), to train and better fit the CNN model for impacted canines using semi-automatically segmented ground truth data; and testing set (n = 50), to test the model performance for AS compared to the ground truth data. Figure 1 illustrates the data distribution for training and testing subsets.

### Data labelling

The ground truth for the training and testing sets was obtained through SS of impacted canines on the online platform using cloud tools such as the contour tool and smart brush function^26^. The contour tool interpolates the interslice region between selected contours, while the smart brush function groups voxels based on their intensities. The operator adjusted the segmentation until satisfied with the result, and all contours were verified in axial, coronal, and sagittal planes. The segmentation was performed by one observer (A.S) and subsequently reassessed by two additional observers (NM & RJ) with 10 and 25 years of experience, respectively. The canines were then exported as standard tessellation language (STL) files for further processing in the CNN pipeline.

### AI architecture

**Figure 2:**
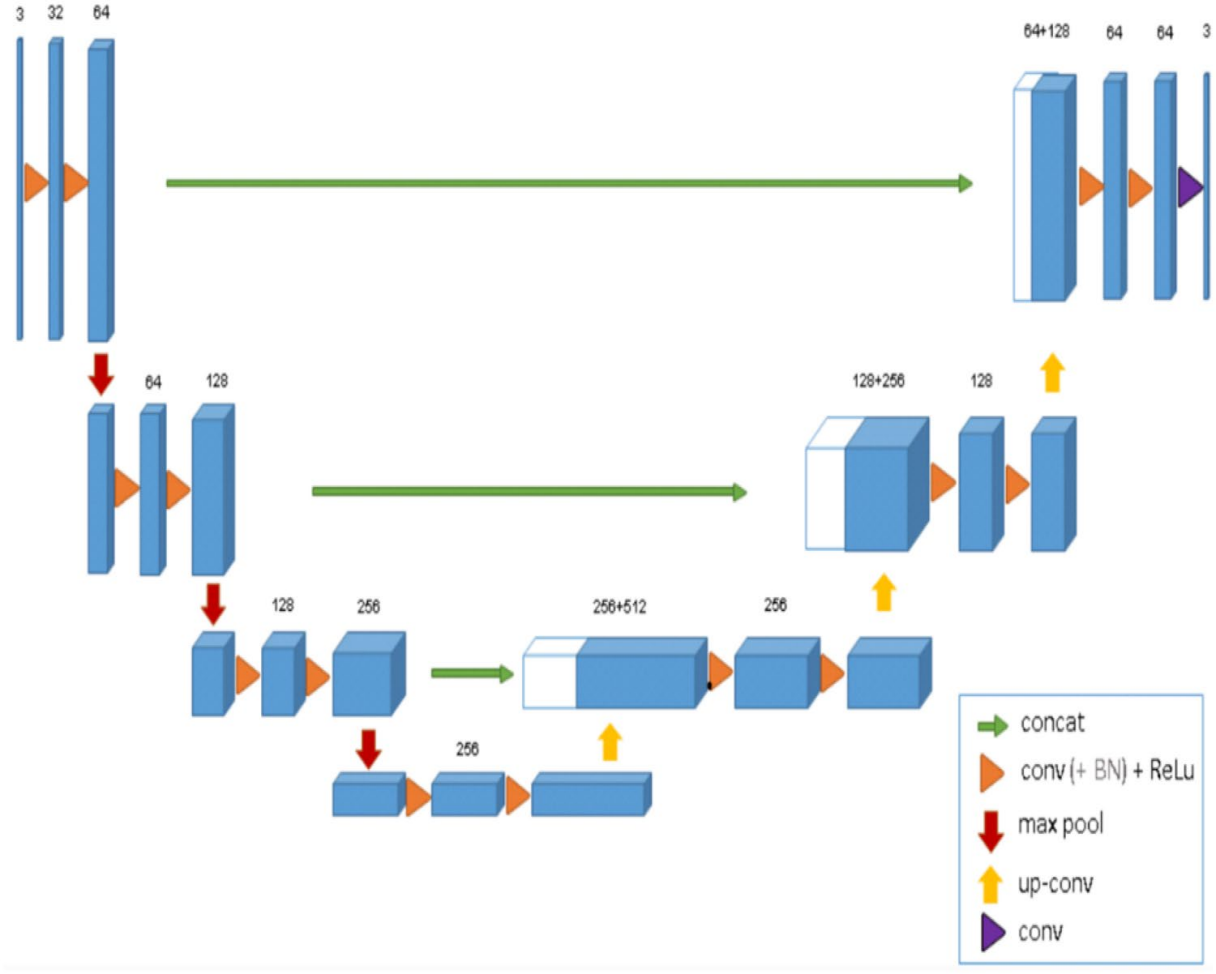
3D U-net architecture^32^.

The training of the CNN model involved the utilization of two 3D U-Net architectures (Fig. 2), each comprising four encoding and three decoding blocks. The architecture included two convolutional layers with a kernel size of 3 × 3 × 3, ReLU activation function, and group normalization with eight feature maps. Max pooling with a kernel size of 2 × 2 × 2 and strides of two was applied to reduce the resolution by a factor of two across all dimensions^25,27^.

Binary classifier training (0 or 1) was performed on both networks. All scans were resampled to a uniform voxel size. To circumvent GPU memory limitations, the entire scan was down-sampled to a fixed size. A low-resolution segmentation was achieved using the first 3D U-Net to propose 3D patches. The segments corresponding to the impacted canines were only extracted. A second 3D U-Net was employed to segment and fuse the relevant patches, which were subsequently used to construct a full-resolution segmentation map. The binary image was binarized, retaining only the largest connected component, and a marching cubes algorithm was applied. The resulting mesh was smoothed to generate a 3D model. The optimization of the model parameters was performed using a deep learning model optimization technique known as ADAM^31^, with an initial learning rate set to 1.25e4. During the training process, random spatial augmentations such as rotation, scaling, and elastic deformation were applied.

### Model testing and consistency of refined segmentations

**Figure 3:**
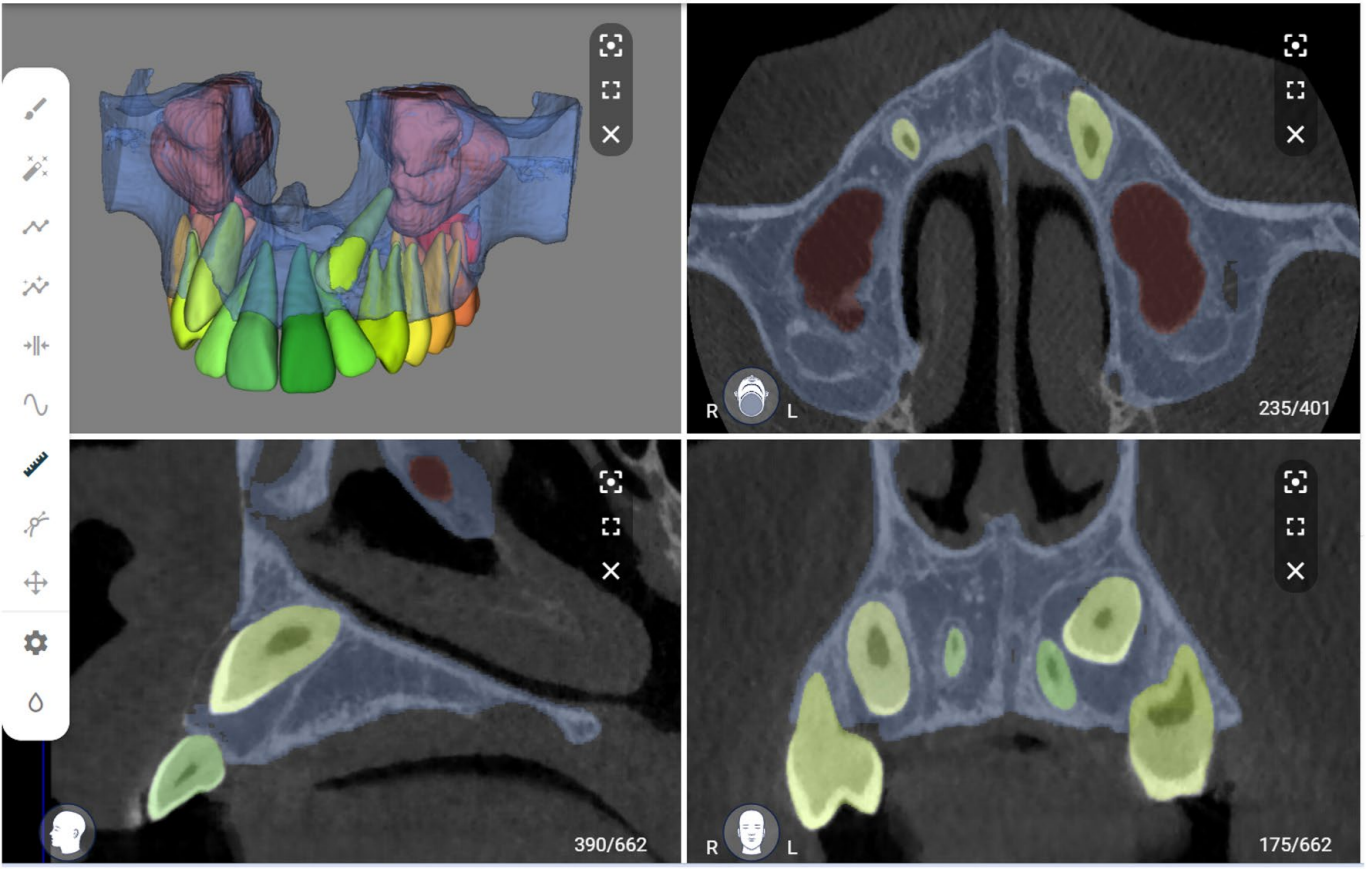
Automated segmentation of maxillary impacted canine and other dentomaxillofacial structures (maxillary bone, maxillary sinus and erupted teeth) on virtual patient creator platform (creator.relu.eu, Relu BV, Version October 2022).

The performance of the CNN model was evaluated using a testing set and compared to the ground truth obtained through SS performed by observer B.E. The images were uploaded to the online tool and the resulting AS was downloaded in STL file format. Moreover, a visual evaluation of the segmented testing set was performed by two observers (A.S, B.E) to determine if any refinements were necessary (Fig. 3). If required, these refinements were implemented using the brush function on the online tool to add or remove voxels from the selection. The refined segmentation was also downloaded in STL file format. The intra- and inter-observer repeatability of refined segmentations was confirmed by both observers performing the refinements twice at a two-week interval.

### CNN performance evaluation

The CNN model’s performance was evaluated based on time duration and voxel- and surface-based metrics.

#### Time analysis

The duration of testing set segmentation with the SS approach was recorded using a digital stopwatch, starting from the import of CBCT data until the generation of the canine model. On the other hand, the online platform automatically provided the time needed to obtain the final segmentation map.

#### Performance metrics

The performance of the CNN model was assessed by utilizing confusion matrix for voxel-wise comparison of SS ground truth and AS maps according to the following metrics: Dice similarity coefficient (DSC), Intersection over union (IoU) and 95% Hausdorff Distance (HD). In addition, the surface-based analysis involved importing superimposed STL files of SS and AS to 3-matic software (Materialise NV, Leuven, Belgium), followed by automated part comparison analysis to calculate the root mean square (RMS) difference between both segmented models.

### Statistical analysis

Data were analyzed using GraphPad Prism, Version 9.0. (GraphPad Software, La Jolla, CA). A paired sample t-test was used to compare the time between SS and AS. The performance metrics were represented by mean and standard deviation values. An IoU score of < 0.5 or HD value of > 0.2 mm would indicate towards poor performance. Inter-class correlation coefficient (r) was applied to assess intra- and inter-observer consistency of the refined segmentations. A p value of less than 0.05 was considered significant.

### Informed consent

Since data were evaluated retrospectively, pseudonymously and were solely obtained for treatment purposes, a requirement of informed consent was waived by the Ethical Review Board of the University Hospitals Leuven (reference number: B322201525552).

## Results

Upon visual inspection of the testing dataset, it was determined that 20% (n = 10) of the cases required minor refinements. The mean values for intra-observer consistency of refinements were 92% for IOU and 96% for DSC. Inter-observer consistency yielded IOU and DSC values of 87% and 93%, respectively (Table 1). Intra-observer repeatability was determined to be 0.992, while inter-observer repeatability was 0.986.

**Table 1:** Intra and inter-observer consistency of refinements.

Metrics	Intra-observer consistency (AS & AS)	Inter-observer consistency (AS & BM)
IOU (intersection over union)		
Mean	0.92	0.87
SD	0.02	0.03
DICE (dice similarity co-efficient)		
Mean	0.96	0.93
SD	0.01	0.02
HD Hausdroff distance (mm)		
Mean	0.09	0.16
SD	0.02	0.03
RMS (root mean square) (mm)		
Mean	0.15	0.23
SD	0.05	0.09

**Figure 4:**
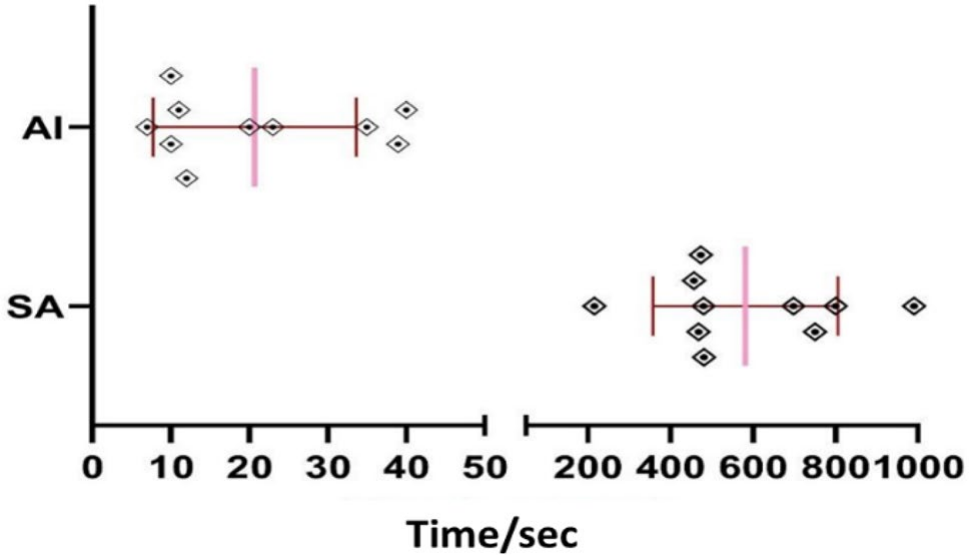
Time comparison between automated and semi-automated segmentation.

The CNN model required an average of 21 s to perform the AS of impacted canines, while the SS took 582 s. This indicates that the CNN model was approximately 24 times faster than the SS method, with a statistically significant difference of (p < 0.0001) (Fig. 4).

**Figure 5:**
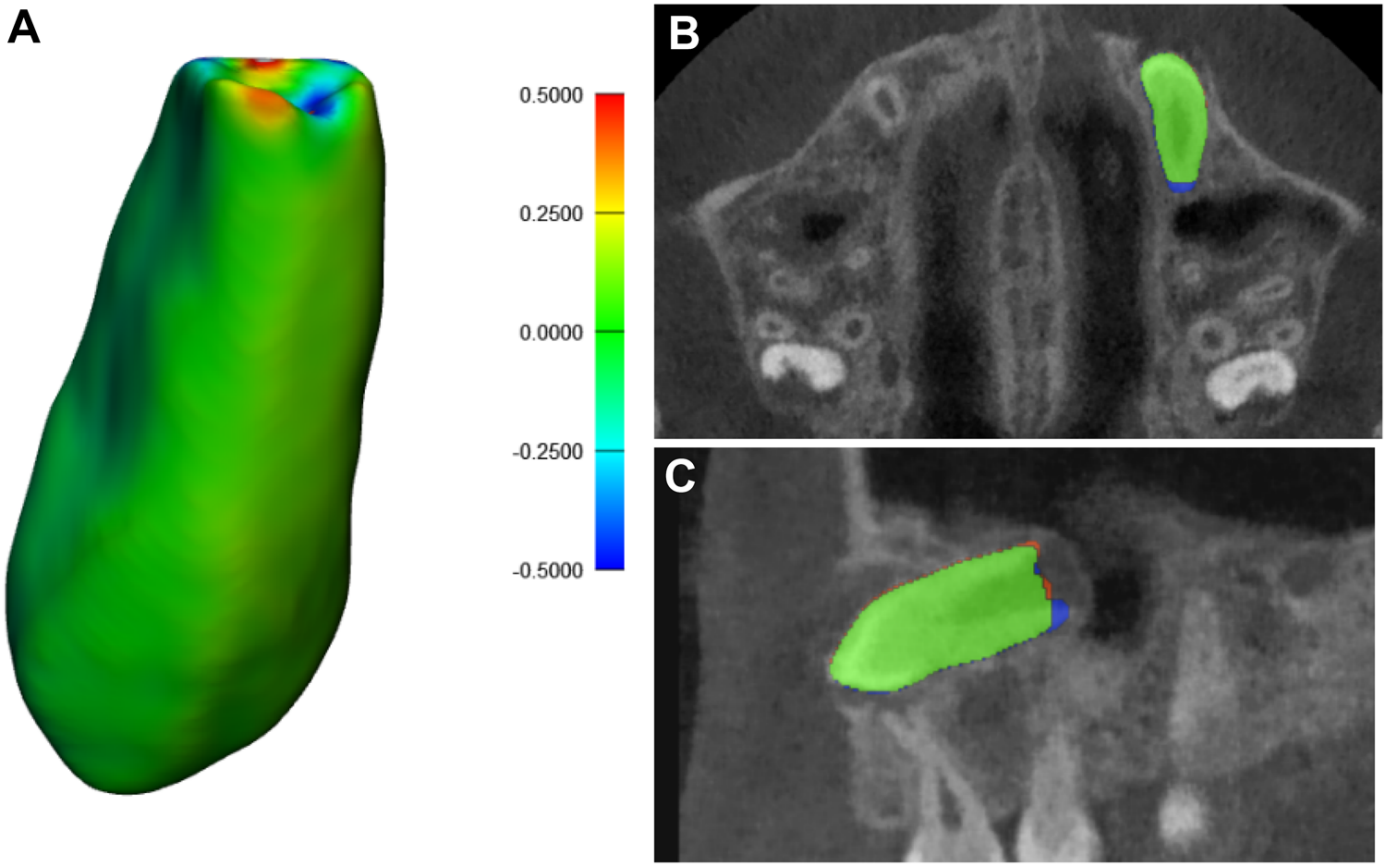
Comparison of automated and semi-automated maxillary impacted canine segmentation. (**A**) three-dimensional surface model. (**B**) axial view. (**C**) sagittal view. Green color corresponds to no difference between automated and semi-automated segmentation surfaces, red color corresponds to overestimation of automated segmentation and blue color corresponds to underestimation of automated segmentation.

The performance metrics of AS demonstrated high values of IoU (0.99 ± 0.04) and DSC (0.99 ± 0.02) when compared to SS. A mean HD value of 0.01 ± 0.03 mm was detected with RMS difference of 0.05 ± 0.25 mm between SS and AS (Table 2 and Fig. 5), hence indicating towards almost a perfect overlap between semi- and fully-automated segmented canine surfaces.

**Table 2:** Performance metrics based on comparison between automated and semi-automated segmentation.

Performance metrics	Mean scoring ± standard deviation
Intersection over union	0.99 ± 0.04
Dice similarity coefficient	0.99 ± 0.02
95% Hausdorff distance(mm)	0.04 ± 0.08
Root mean square difference (mm)	0.05 ± 0.25

## Discussion

A precise 3D segmentation of impacted canine is essential mainly for digital orthodontic treatment planning workflows^33–35^. Despite being a challenging and time-consuming task through manual and semi-automated approaches, CNN-based automation has the ability to produce highly accurate 3D virtual models in a time-efficient manner^22,24,28^. Hence, the goal of this study was to introduce and assess the performance of a CNN model for the segmentation of maxillary impacted canines. In this study, we utilized a pre-existing cloud-based platform that had been previously trained to segment multiple dentomaxillofacial structures (permanent teeth, maxillary sinus, inferior alveolar nerve, and jaw bones) and apply automated CBCT-intraoral scan registration. The performance of the model was comparable to that of SS performed by clinical experts. It is noteworthy that the model showed 100% consistency without the issue of human variability, where it was able to produce identical results when segmenting the same case multiple times. Moreover, only minor refinements were required which confirmed high similarity between AS and SS.

The presented CNN model was able to automatically segment impacted canines in 21 s, which was 24 times faster than the SS approach. Hence, demonstrating the benefits of incorporating automation into the digital workflow to increase clinical efficiency. A comparison with existing studies regarding time-efficiency was challenging due to a lack of reported time data. Time is a crucial factor in clinical dentistry and is integral to an optimal digital workflow, hence it should be reported in such studies incorporating artificial intelligence (AI) based solutions.

Limited research has been conducted on the application of deep learning-based CNNs for either classification or segmentation of impacted teeth. Specifically, no studies have focused on impacted canine segmentation on CBCT images. Hence, comparison with existing evidence was deemed difficult. Kuwada et al.^36^ evaluated the performance of three CNN systems (DetectNet, VGG-16, AlexNet) for detecting and classifying maxillary impacted supernumerary teeth on panoramic images. They found that DetectNet had the highest detection performance with a recall and precision of 1. Celik et al.^37^ proposed a deep learning-based tool for detecting impacted mandibular third molars. They compared a two-stage technique (Faster RCNN with ResNet50, AlexNet, and VGG16 as backbones) with a one-stage technique (YOLOv3) and found that YOLOv3 had the highest detection efficacy with an average precision of 0.96. Imak et al.^38^ used ResMIBCU-Net to segment impacted teeth (including impacted canines) on panoramic images and achieved an accuracy of 99.82%. Orhan et al.^39^ evaluated the diagnostic performance of a U-Net CNN model for detecting impacted third molar teeth on CBCT images and showed an accuracy of 86.2%. Meanwhile, the findings of the present suggested a high scoring of 0.99 based on both DSC and IoU. It is noteworthy that the use of accuracy as an evaluation metric for 3D AS tasks can result in misleading conclusions due to the inclusion of true negatives in the calculation. This phenomenon, known as the accuracy paradox, can result in a high accuracy value despite poor model performance^40^. This is particularly evident in imbalanced datasets where the over-representation of one class can lead to an overestimation of accuracy. Alternative evaluation metrics, such as, DSC, 95% HD and IoU should provide a more optimal representation of model performance.

The study’s main strength was its ability to accurately and rapidly segment impacted canines with various angulations (horizontal, oblique, vertical) on CBCT images. The inclusion of scans from two CBCT devices with different acquisition parameters and metal artifacts from brackets could enhance the tool’s practicality and robustness. Moreover, the segmentation and refinements could be performed on an easily accessible online platform without the need for third-party software, making it more convenient for clinical use.

The study also had certain limitations. Firstly, the training was limited to only maxillary impacted canines without inclusion of any other impactions. Secondly, the online tool only provided the segmentation map as an outcome without any additional tools for dimensional and morphometric measurements. Thirdly, the CNN training was based on two CBCT devices. Further studies are warranted to train the model based on the datasets from multiple CBCT devices with different scanning parameters and qualities, as well as images acquired from different institutions, for justifying its applicability for regular clinical tasks.

## Conclusion

The proposed CNN model facilitated a rapid, consistent, and precise segmentation of maxillary impacted canines on CBCT images, which might aid in diagnosis and the planning of orthodontic and oral surgical interventions. The integration of impacted canine segmentation into the online tool could be considered as a significant leap towards achieving a fully AI assisted virtual workflow for planning, surgical guide designing, and follow-up assessment for various dentomaxillofacial procedures.

## Data Availability

The data analyzed during the current study available from the corresponding author on reasonable request.
